# High *AHR* expression in breast tumors correlates with expression of genes from several signaling pathways namely inflammation and endogenous tryptophan metabolism

**DOI:** 10.1371/journal.pone.0190619

**Published:** 2018-01-10

**Authors:** Sophie Vacher, Patrice Castagnet, Walid Chemlali, François Lallemand, Didier Meseure, Marc Pocard, Ivan Bieche, Martine Perrot-Applanat

**Affiliations:** 1 Department of Genetics, Pharmacogenomics Unit, Institut Curie, Paris, France; 2 Department of Pathology, Lariboisière-Saint Louis Hospital, Paris, France; 3 Department of Pathology, Institut Curie, Paris, France; 4 INSERM U965, Lariboisière-Saint Louis Hospital, Paris, France; 5 University of Paris Diderot-Paris 7, Paris, France; 6 EA7331, University of Paris Descartes, Paris, France; Lawrence Berkeley National Laboratory, University of California, Berkeley, UNITED STATES

## Abstract

Increasing epidemiological and animal experimental data provide substantial support for the role of aryl hydrocarbon receptor (AhR) in mammary tumorigenesis. The effects of AhR have been clearly demonstrated in rodent models of breast carcinogenesis and in several established human breast cancer cell lines following exposure to AhR ligands or AhR overexpression. However, relatively little is known about the role of AhR in human breast cancers. AhR has always been considered to be a regulator of toxic and carcinogenic responses to environmental contaminants such as TCDD (dioxin) and benzo[a]pyrene (BaP). The aim of this study was to identify the type of breast tumors (ERα-positive or ERα-negative) that express *AHR* and how AhR affects human tumorigenesis. The levels of *AHR*, *AHR* nuclear translocator (*ARNT*) and *AHR* repressor (*AHRR*) mRNA expression were analyzed in a cohort of 439 breast tumors, demonstrating a weak association between high *AHR* expression and age greater than fifty years and ERα-negative status, and HR-/ERBB2 breast cancer subtypes. *AHRR* mRNA expression was associated with metastasis-free survival, while *AHR* mRNA expression was not. Immunohistochemistry revealed the presence of AhR protein in both tumor cells (nucleus and/or cytoplasm) and the tumor microenvironment (including endothelial cells and lymphocytes). High *AHR* expression was correlated with high expression of several genes involved in signaling pathways related to inflammation (*IL1B*, *IL6*, *TNF*, *IL8* and *CXCR4*), metabolism (*IDO1* and *TDO2* from the kynurenine pathway), invasion (*MMP1*, *MMP2* and *PLAU*), and IGF signaling (*IGF2R*, *IGF1R* and *TGFB1*). Two well-known ligands for *AHR* (TCDD and BaP) induced mRNA expression of *IL1B* and *IL6* in an ERα-negative breast tumor cell line. The breast cancer ER status likely influences AhR activity involved in these signaling pathways. The mechanisms involved in AhR activation and target gene expression in breast cancers are also discussed.

## Introduction

Breast cancer is the most common cancer in women in Western countries. All of the established risk factors combined can only explain less than half of all cases of breast cancer [1–2]. Environmental factors, *i*.*e*. the world around us and the way we live today, are probably also involved. Of particular concern are pollutants that alter the endocrine system and may modify cancer risks [3–6]. Epidemiological studies conducted after the “Seveso accident”, one of the best known industrial accidents, and studies on chemical workers exposed to 2,3,7,8-tetrachlorodibenzo-p-dioxin (TCDD or dioxin) [6] have revealed increased risks of developing breast cancer following exposure to TCDD, a potent ligand of the aryl hydrocarbon nuclear receptor (AhR). In particular, breast cancer incidence increased even more than 15 and 30 years after the Seveso accident [7–9]. Growing evidence from animal studies, including those performed on TCDD-exposed rodents during gestation, and on a number of established human breast cancer cell lines, provides substantial support for the role of AhR in mammary tumorigenesis. However, relatively little is known about the role of AhR in human primary breast tumors.

Over recent decades, AhR has been characterized as a regulator of toxic and carcinogenic responses to environmental AhR xenobiotics, such as TCDD, polycyclic aromatic hydrocarbons and halogenated aromatic hydrocarbons [10]. These ligands are widespread environmental contaminants. The repertoire of AhR ligands has been considerably expanded and now includes many industrial compounds, several classes of chemoprotective phytochemicals, namely flavonoids (quercetin), several indoles [(indole-3-carbinol, FICZ (6-formylindolo[3,2b]carbazole)], and several pharmaceuticals (omeprazole, hydroxytamoxifen, tranilast, aminoflavone) [11]. No study has addressed the molecular consequences of a combination of environmental and dietary ligands (a situation that is likely to occur in the environment) with an endogenous tumor-promoting AhR ligand [12].

Our understanding of AhR function was initially derived from toxicology and pharmacology studies on the response of AhR to xenobiotics, including regulation of the expression of xenobiotic metabolizing enzymes such as Cyp1A1 and Cyp1B1 [13–14]. More recent studies have demonstrated that AhR may be involved in other important cellular and pathological processes such as control of proliferation, regulation of cell cycle and cell migration [15], angiogenesis [16] and tumorigenesis [17–19]. Importantly, *AHR* modulates the response of immune cells to the presence of environmental and endogenous compounds [20–22]. These functions are likely dependent upon ligand-mediated activation of the receptor. The molecular pathway leading to AhR activation by exogenous ligands has been extensively studied. Once activated by ligands, AhR translocates into the nucleus and forms heterodimers with the AhR nuclear translocator protein (ARNT) to activate downstream gene transcription through interactions with cognate dioxin-responsive elements (DREs) located on AhR-responsive gene promoters (reviewed in [11]). An AhR repressor (AhRR) has been identified that regulates AhR activity by binding and sequestering the nuclear translocator ARNT [23–24]. More recent findings suggest that AhR may also participate in cross-talk with other transcription factors such as estrogen receptor (ERα) and NF-KB [25–27].

Various animal experimental data have provided substantial support for an association between abnormal AhR expression/function and breast cancer [4,17,28–30]. TCDD is the prototypical and most potent known environmental AhR ligand. Its effects on breast cancer were first reported in rodent models of tumorigenesis, including TCDD-induced breast tumors that express high levels of *AHR* [17,29–30]. The effect of different concentrations and the timing of exposure to TCDD on tumor development have been reported [4,28]. Studies have also focused on using cancer cells as models to determine the mechanisms and pathways activated by TCDD and other AhR ligands. Upregulation of *AHR* expression and transcriptional activity plays an important role in several, if not all, stages of malignant transformation [31–32]. Knock-down of aberrantly upregulated aryl hydrocarbon receptor reduces tumor growth and metastasis of MDA-MB-231 [33]. However, activation of AhR inhibits invasive and metastatic features of human breast cancer cells and promotes breast cancer cell differentiation [34]. Immortalized mammary fibroblasts lacking AhR also have impaired tumorigenic potential in a subcutaneous mouse xenograft model [35]. Different responses in sensitive genes have been observed in different breast cancer cells [36]. Recent studies indicate that AhR could also play distinct roles in the survival, migration and invasion of ERα-positive as compared to ERα-negative breast tumor cell lines in the absence of environmental chemicals [12,37]. A high degree of complexity has therefore emerged concerning the role of AhR in mammary tumorigenesis in various *in vitro* systems.

Despite clear-cut demonstration of the role of AhR in rodent models of carcinogenesis and in a number of established human breast cancer cell lines, relatively little is known about the roles of AhR in human primary breast tumors [19,38]. In particular, it is not known whether the relative expression of AhR may be a determinant factor for its role in breast tumor development, and very few studies have been published on the nature of AhR-positive cells in breast tumors or the abundance and potency of AhR ligands within the tumor [39]. The primary objectives of this study were therefore to identify the types of breast tumors that express *AHR* and investigate whether variations in *AHR* gene expression are associated with classical pathological parameters and outcome and with a panel of other gene expressions in order to provide insight into the signaling pathways that correlate with *AHR* expression levels, and thus affect breast tumor growth and progression.

## Material and methods

### Patients and samples for analysis of *AHR* and *AHRR* expression

Samples of 439 primary unilateral invasive breast tumors excised from women managed at the Institut Curie-René Huguenin Hospital (Saint-Cloud, France) between 1978 and 2008 were analyzed. Each patient signed a written informed consent form and this study was approved by the Institut Curie-René Huguenin Hospital Ethics Committee. Patients were followed at the Institut Curie-René Huguenin Hospital.

Patients had a mean age of 61.8 years (range: 31–91 years) and had to meet the following inclusion criteria: a) primary unilateral nonmetastatic breast carcinoma for which complete clinical, histological and biological data were available; b) no radiotherapy or chemotherapy prior to surgery. Treatment consisted of radical mastectomy in 277 cases (63.5%) and breast-conserving surgery plus locoregional radiotherapy in 159 cases (36.5%) (information available for 436 cases). Tumor samples were collected immediately after biopsy or surgery and were stored in liquid nitrogen until mRNA extraction.

Patient follow-up consisted of physical examination and routine chest radiography every 3 months for 2 years, and annually thereafter. Mammograms were performed annually. Adjuvant therapy was administered to 354 patients, consisting of chemotherapy alone in 86 cases, hormone therapy alone in 168 cases, and combined chemotherapy and hormone therapy in 100 cases.

The histological type and the number of positive axillary nodes were established at the time of surgery. The malignancy of infiltrating carcinomas was scored according to the Scarff-Bloom-Richardson (SBR) histoprognostic system. Hormone receptor (HR) [estrogen receptor alpha (ERα), progesterone receptor (PR)] and human epidermal growth factor receptor 2 (ERBB2) protein status was determined at by biochemical methods (dextran-coated charcoal method, enzyme immunoassay or immunohistochemistry) [40] and was confirmed by real-time quantitative RT-PCR assay.

Patients were divided into four groups according to HR (ERα and PR) and ERBB2 status, as follows: two luminal subtypes [HR+ (ERα+ or PR+)/ERBB2+ (n = 50)] and [HR+ (ERα+ or PR+)/ERBB2- (n = 281)]; ERBB2+ subtype [HR- (ERα- and PR-) /ERBB2+ (n = 41)], and triple-negative subtype [HR- (ERα- and PR-) /ERBB2- (n = 67)]. Median follow-up was 9.1 years (range: 130 days to 33 years) and 172 patients developed metastasis during follow-up. Clinical and pathological characteristics of patients in relation to metastasis-free survival (MFS) are provided in Table 1. Seven specimens of adjacent “normal” breast tissue from breast cancer patients or normal breast tissue from women undergoing cosmetic breast surgery were used as sources for normal mRNA.

**Table 1:** Clinical and pathological characteristics of patients in relation to metastasis-free survival (MFS).

	Number of patients (%)	Number of metastases (%)	MFS *p*-value ^a^
*Total*	439 (100)	172 (39.2)	
*Age*			
≤50	93 (21.2)	36 (38.7)	0.89 (NS)
>50	346 (78.8)	136 (39.3)	
*SBR histological grade* ^b^^,^ ^c^			
I	57 (13.3)	11 (19.3)	**0.0017**
II	218 (50.7)	87 (39.9)	
III	155 (36.0)	70 (45.2)	
*Lymph node status* ^d^			
0	116 (26.7)	35 (30.2)	**0.00000059**
1–3	227 (52.2)	78 (34.4)	
>3	92 (21.1)	57 (62.0)	
*Macroscopic tumor size* ^e^			
≤25mm	214 (49.7)	64 (29.9)	**0.000013**
>25mm	217 (50.3)	107 (49.3)	
*ERα status*			
Negative	113 (25.7)	46 (40.7)	0.19 (NS)
Positive	326 (74.3)	126 (38.7)	
*PR status*			
Negative	187 (42.6)	81 (43.3)	**0.029**
Positive	252 (57.4)	91 (36.1)	
*ERBB2 status*			
Negative	348 (79.3)	134 (38.5)	0.50 (NS)
Positive	91 (20.7)	38 (41.8)	
*Molecular subtypes*			
HR+ ERBB2+	50 (11.4)	18 (36.0)	0.17 (NS)
HR+ ERBB2-	281 (64.0)	109 (38.8)	
HR- ERBB2+	41 (9.3)	20 (48.8)	
HR- ERBB2-	67 (15.3)	25 (37.3)	
*PIK3CA mutation status*			
wild type	293 (66.7)	121 (41.3)	0.11 (NS)
mutated	146 (33.3)	51 (34.9)	

Abbreviations: ERα: estrogen receptor alpha; PR: progesterone receptor; ERBB2: human epidermal growth factor receptor 2; HR: hormone receptor.

Values shown in bold type are statistically significant (*p*-value<0.05). NS: not significant.

^a^ Log-rank test.

^b^ Scarff-Bloom-Richardson classification.

^c^ Information available for 430 patients.

^d^ Information available for 435 patients.

^e^ Information available for 431 patients.

### Real-time qRT-PCR expression of *AHR* and *AHRR* genes, and genes involved in *AHR* signaling pathways

The conditions of total RNA extraction, complementary DNA synthesis and qRT-PCR were as described in protocols.io: http://dx.doi.org/10.17504/protocols.io.kehctb6.

Results, expressed as N-fold differences in target gene expressions relative to the *TBP* gene (and termed “Ntarget”), were determined as Ntarget = 2^ΔCtsample^, where the ΔCt value of the sample was determined by subtracting the Ct value of the specific target gene from the Ct value of the *TBP* gene. Ntarget values of the samples were subsequently normalized so that the median of Ntarget values for normal breast tissues was 1 for *AHR* and *AHRR* expressions, and so that the value for “basal mRNA level” (smallest amount of quantifiable target gene mRNA, Ct = 35) was 1 for AHR signaling pathway genes.

The nucleotide sequences of the primers used were as follows: *AHR*-U (5’-TAA CCC AGA CCA GAT TCC TCC AGA-3’) and *AHR*-L (5’-CCC TTG GAA ATT CAT TGC CAG A -3’) for *AHR* gene (PCR product of 115 bp), *AHRR*-U (5’-GGA AGG CTG CTG TTG GAG TCT CT-3’) and *AHRR*-L (5’- TGG AAG CCC AGA TAG TCC ACG A-3’) for *AHRR* gene (PCR product of 104 bp), and *TBP*-U (5’-TGC ACA GGA GCC AAG AGT GAA-3’) and *TBP*-L (5’-CAC ATC ACA GCT CCC CAC CA-3’) for *TBP* gene (PCR product of 132 bp). Primers of the 54 additional genes tested in this study are available on request.

### Cell culture

MDA-MB-436 cells, an ERα-negative cell line purchased from ATCC, were maintained in DMEM medium containing 5% foetal bovine serum (Invitrogen, Carlsbad, CA) and 1% antibiotics (50 μg/mL penicillin, 50 μg/mL streptomycin, 100 μg/mL neomycin), and grown at 37°C in a humidified atmosphere of 5% (v/v) CO_2_ in air. Two *AHR* ligands [TCDD (2,3,7,8-tetrachlorodibenzo-p-dioxin) and BaP (benzo[a]pyrene)], purchased from Sigma-Aldrich (St-Quentin Fallavier, France) were used. T-test was used to compare the treated groups with the control group.

### Immunohistochemistry

Immunohistochemical labeling was performed as previously described [5]. Immunohistochemical staining for AhR (rabbit polyclonal antibody clone H211, Santa Cruz, 1/50 dilution) was performed using Ventana Autostainer (USA). Sections of some tumors were also immunostained with antibodies directed against Cyp1B1 (Santa Cruz, dilution 1/200) and CD4 (clone Sp35, Roche Ventana, USA). The antigen-antibody complex was visualized using DAB as chromogen, as previously described [5]. AhR immunostaining was analyzed blindly in duplicate by two specialists including a certified pathologist [5].

### Statistical analysis

The relative expression of each gene was characterized by the median and the range, as described above. Relationships between *AHR* or *AHRR* mRNA expression and clinical parameters were identified using non-parametric tests, the Kruskal-Wallis H test (relationship between one quantitative parameter and two or more qualitative parameters) and Spearman’s rank correlation test (relationship between two quantitative parameters). Differences were considered significant at confidence levels greater than 95% (p<0.05). To visualize the efficacy of a molecular marker to discriminate between two populations (patients who developed/or who did not develop metastases) in the absence of an arbitrary cut-off value, data were summarized in a ROC (receiver operating characteristic) curve [41]. The AUC (area under the curve) was calculated as a single measure to discriminate efficacy. Survival distributions were estimated by the Kaplan-Meier method, and the significance of differences between survival rates was ascertained with the log-rank test. Metastasis-free survival (MFS) was determined as the interval between initial diagnosis and detection of the first metastasis. Cox’s proportional hazards regression model was used to assess prognostic significance in multivariate analysis [42].

## Results

### Relationships between *AHR* mRNA expression in breast tumors and classical clinical and pathological parameters and patient outcome

*AHR* mRNA expression level was assessed in the cohort of 439 tumor specimens and was compared to normal breast tissue samples (Table 2). A fairly wide range of *AHR* mRNA expression was observed (0.0 to 5.77). *AHR* mRNA expression level was weakly associated only with one classical prognostic factor, *i*.*e*. age (p = 0.040). The cohort (n = 439) was classified into four breast cancer subtypes on the basis of hormone receptor (ERα and PR) and ERBB2 status: HR+/ERBB2+ (n = 50), HR+/ERBB2- (n = 281), HR-/ERBB2+ (n = 41) and HR-/ERBB2- (n = 67). *AHR* mRNA expression level was weakly associated with ERα and HR negative status (p = 0.039 and p = 0.018, respectively) and HR-/ERBB2- breast cancer subtypes (p = 0.047) (Table 2). The possible relationship between *AHR* and *MKI67* expression, *EGFR* expression or *PIK3CA* mutation status was also tested in the same tumors. No correlation was observed between *AHR* and *MKI67* or *EGFR* mRNA levels (Spearman’s rank correlation coefficient: r = +0.082, p = 0.082; r = +0.027, p = 0.58, respectively), and between *AHR* mRNA levels and *PIK3CA* mutation status (p = 0.32) (Table 2).

**Table 2:** Relationship between *AHR* mRNA expression and classical clinical and pathological parameters in a series of 439 breast cancers.

	Total population (%)	*AHR* mRNA expression relative to normal breast	*p*-value ^a^
*Total*	439 (100.0)	0.50 (0.00–5.77)	
*Age*			
≤50	93 (21.2)	0.41 (0.00–3.15)	**0.040**
>50	346 (78.8)	0.54 (0.00–5.77)	
*SBR histological grade* ^b^^,^ ^c^			
I	57 (13.3)	0.31 (0.00–3.02)	0.070 (NS)
II	218 (50.7)	0.52 (0.00–5.77)	
III	155 (36.0)	0.53 (0.00–2.84)	
*Lymph node status* ^d^			
0	116 (26.7)	0.54 (0.00–3.69)	0.30 (NS)
1–3	227 (52.2)	0.52 (0.00–5.77)	
>3	92 (21.1)	0.47 (0.00–2.96)	
*Macroscopic tumor size* ^e^			
≤25mm	214 (49.7)	0.48 (0.00–5.77)	0.48 (NS)
>25mm	217 (50.3)	0.53 (0.00–4.88)	
*ERα status*			
Negative	113 (25.7)	0.56 (0.03–2.96)	**0.039**
Positive	326 (74.3)	0.47 (0.00–5.77)	
*PR status*			
Negative	187 (42.6)	0.54 (0.00–3.69)	0.068 (NS)
Positive	252 (57.4)	0.47 (0.00–5.77)	
*HR status*			
Negative	108 (24.6)	0.57 (0.03–2.96)	**0.018**
Positive	331 (75.4)	0.47 (0.00–5.77)	
*ERBB2 status*			
Negative	348 (79.3)	0.51 (0.00–5.77)	0.47 (NS)
Positive	91 (20.7)	0.48 (0.00–3.15)	
*Molecular subtypes*			
HR+ ERBB2+	50 (11.4)	0.39 (0.00–3.15)	**0.047**
HR+ ERBB2-	281 (64.0)	0.48 (0.00–5.77)	
HR- ERBB2+	41 (9.3)	0.56 (0.03–2.40)	
HR- ERBB2-	67 (15.3)	0.60 (0.07–2.96)	
*PIK3CA mutation status*			
wild type	293 (66.7)	0.50 (0.00–5.77)	0.32 (NS)
mutated	146 (33.3)	0.50 (0.00–3.65)	
*MKI67 mRNA expression*			
Median	12.2 (0.80–117)	0.50 (0.00–5.77)	r = +0.082 ^f^
			p = 0.082 (NS)
*EGFR mRNA expression*			
median	0.21 (0.00–106)	0.50 (0.00–5.77)	r = +0.027 ^f^
			p = 0.58 (NS)

Abbreviations: ERα: estrogen receptor alpha; PR: progesterone receptor; HR: hormone receptor; ERBB2: human epidermal growth factor receptor 2; PIK3CA: phosphatidylinositol-4,5-bisphosphate 3-kinase catalytic subunit alpha; MKI67: marker of proliferation Ki-67; EGFR: epidermal growth factor receptor.

Values shown in bold type are statistically significant (*p*-value<0.05). NS: not significant.

^a^ Kruskal-Wallis H Test.

^b^ Scarff-Bloom-Richardson classification.

^c^ Information available for 430 patients.

^d^ Information available for 435 patients.

^e^ Information available for 431 patients.

^f^ Spearman’s rank correlation.

The impact of *AHR* mRNA levels on patient outcome was also assessed by studying MFS survival curves. AUC analyses were performed to identify a cut-off point, which divides the cohort into relevant *AHR* expression subgroups. No link was observed between *AHR* mRNA levels and MFS, suggesting that *AHR* expression is not a prognostic factor in breast cancer (data not shown).

### Relationship between *AHRR* mRNA expression in breast tumors and classical clinical and pathological parameters and patient outcome

*AHRR* functions as a feedback modulator by repressing *AHR*-dependent gene expression, and may therefore have a major impact on the AHR signaling pathway [23,43]. Consequently, *AHRR* mRNA expression was also assessed in the cohort of 439 samples and was compared to normal breast tissue samples (Table 3). A very wide range of *AHRR* mRNA expression was also observed (0.0 to 19.8).

**Table 3:** Relationship between *AHRR* mRNA expression and classical clinical and pathological parameters in a cohort of 439 breast cancers.

	Total population (%)	*AHRR* mRNA expression relative to normal breast	*p*-value ^a^
*Total*	439 (100.0)	1.40 (0.00–19,8)	
*Age*			
≤50	93 (21.2)	1.54 (0.00–11.3)	0.22 (NS)
>50	346 (78.8)	1.35 (0.00–19.8)	
*SBR histological grade* ^b^^,^ ^c^			
I	57 (13.3)	1.32 (0.19–5.35)	0.47 (NS)
II	218 (50.7)	1.38 (0.00–17.9)	
III	155 (36.0)	1.46 (0.00–19.8)	
*Lymph node status* ^d^			
0	116 (26.7)	1.47 (0.00–13.2)	0.29 (NS)
1–3	227 (52.2)	1.31 (0.00–19.8)	
>3	92 (21.1)	1.42 (0.00–11.3)	
*Macroscopic tumor size* ^e^			
≤25mm	214 (49.7)	1.31 (0.00–19.8)	0.34 (NS)
>25mm	217 (50.3)	1.45 (0.00–17.9)	
*ERα status*			
Negative	113 (25.7)	1.36 (0.00–19.8)	0.67 (NS)
Positive	326 (74.3)	1.40 (0.00–17.9)	
*PR status*			
Negative	187 (42.6)	1.27 (0.00–19.8)	0.37 (NS)
Positive	252 (57.4)	1.42 (0.00–17.9)	
*HR status*			
Negative	108 (24.6)	1.29 (0.00–19.8)	0.84 (NS)
Positive	331 (75.4)	1.40 (0.00–17.9)	
*ERBB2 status*			
Negative	348 (79.3)	1.42 (0.00–19.8)	0.34 (NS)
Positive	91 (20.7)	1.25 (0.00–13.2)	
*Molecular subtypes*			
HR+ ERBB2+	50 (11.4)	1.46 (0.00–9.87)	0.16 (NS)
HR+ ERBB2-	281 (64.0)	1.40 (0.00–17.9)	
HR- ERBB2+	41 (9.3)	1.11 (0.00–13.2)	
HR- ERBB2-	67 (15.3)	1.65 (0.00–19.8)	
*PIK3CA mutation status*			
wild type	293 (66.7)	1.43 (0.00–19.8)	0.27 (NS)
Mutated	146 (33.3)	1.35 (0.00–13.2)	
*MKI67 mRNA expression*			
median	12.2 (0.80–117)	1.40 (0.00–19.8)	r = +0.131 ^f^
			p = **0.0058**
*EGFR mRNA expression*			
Median	0.21 (0.00–106)	1.40 (0.00–19.8)	r = +0.007 ^f^
			p = 0.89 (NS)
*AHR mRNA expression*			
median	0.50 (0.00–5.77)	1.40 (0.00–19.8)	r = +0.115 ^f^
			p = **0.015**

Abbreviations: ERα: estrogen receptor alpha; PR: progesterone receptor; HR: hormone receptor; ERBB2: human epidermal growth factor receptor 2; PIK3CA: phosphatidylinositol-4,5-bisphosphate 3-kinase catalytic subunit alpha; *MKI67*: marker of proliferation Ki-67; *EGFR*: epidermal growth factor receptor; *AHR*: aryl hydrocarbon receptor.

Values shown in bold type are statistically significant (*p*-value<0.05). NS: not significant.

^a^ Kruskal-Wallis H Test.

^b^ Scarff-Bloom-Richardson classification.

^c^ Information available for 430 patients.

^d^ Information available for 435 patients.

^e^ Information available for 431 patients.

^f^ Spearman’s rank correlation.

The level of *AHRR* mRNA expression was not associated with any classical clinical and pathological factors, *i*.*e*. age, SBR histological grade, lymph node status, macroscopic tumor size or breast cancer subtype. No correlation was observed between *AHRR* and *EGFR* mRNA expression, or between *AHRR* mRNA expression and *PIK3CA* mutation status (Table 3). *AHRR* mRNA expression was only positively correlated with *MKI67* mRNA expression (Spearman’s rank correlation coefficient: r = +0.131, p = 0.0058) (Table 3), suggesting a link between *AHRR* mRNA expression and cell proliferation. The level of *AHRR* mRNA expression was also correlated with *AHR* mRNA levels (Spearman’s rank correlation coefficient: r = +0.115, p = 0.015) (Table 3).

**Fig 1:**
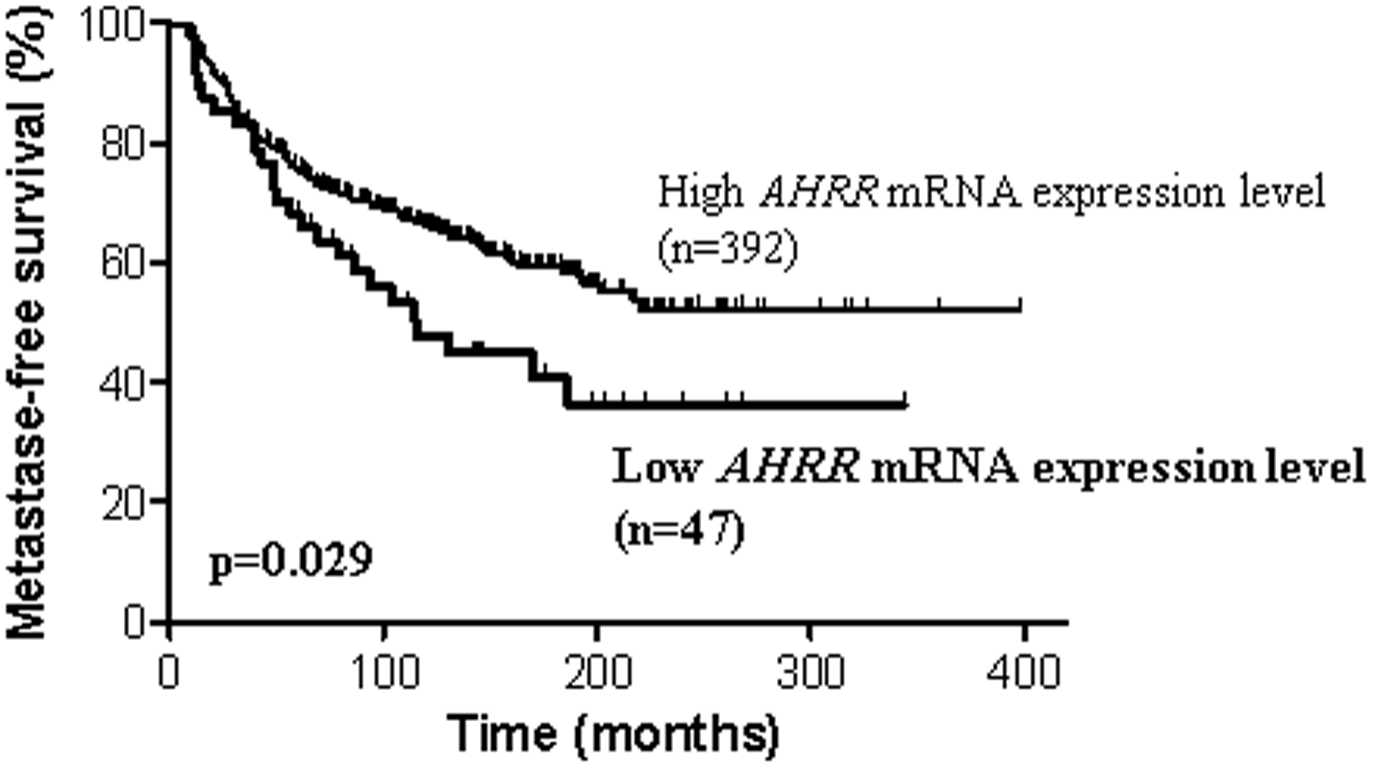
Survival curves of two groups of patients according to *AHRR* mRNA expression level in the cohort of 439 breast tumors. AUC analysis was used to divide the population into two relevant *AHRR* expression subgroups.

Tumors with the lowest levels of *AHRR* mRNA expression (≤0.49, n = 47, 10.7%) were significantly associated with poor MFS (p = 0.029; Fig 1), compared to tumors with higher levels of *AHRR* mRNA expression (>0.49, n = 392, 89.3%). Patients with the lowest levels of *AHRR* mRNA expression had a 5-year MFS of 68.1% +/- 6.8% and a 10-year MFS of 47.7% +/- 7.73%. Patients with the highest levels of *AHRR* mRNA expression had a 5-year MFS of 75.3% +/- 2.2% and a 10-year MFS of 66.4% +/- 2.49%. Multivariate analysis using a Cox proportional hazards model assessed the prognostic value for MFS of the parameters found to be significant on univariate analysis, i.e., SBR histological grade, lymph node status, macroscopic tumor size, PR status (Table 1) and *AHRR* mRNA level. The prognostic significance of lymph node status (p = 0.00025), macroscopic tumor size (p = 0.0062), SBR histological grade (p = 0.011) and *AHRR* mRNA level (p = 0.033) was maintained. This analysis confirmed that *AHRR* mRNA level is an independent prognostic factor in breast cancer.

### mRNA expression analysis of genes involved in the *AHR* signaling pathway in the ERα-negative and ERα-positive subpopulations

The ER status of breast cancer cells and tumors is known to influence AHR transcriptional activity [36,44]. In order to study the possible implication of *AHR* expression on the expression of genes involved in the AHR signaling pathway regardless of ERα status, mRNA levels of 54 candidate genes were therefore analyzed in the two subpopulations (ERα-positive and ERα-negative) of this cohort of 439 breast cancers: 26 low *AHR*-expressing and 26 high *AHR*-expressing ERα-negative breast tumors, and 14 low *AHR*-expressing and 14 high *AHR*-expressing ERα-positive breast tumors. The cut-off to distinguish high *AHR* expression from low *AHR* expression in the two subpopulations (ERα-positive and ERα-negative) was defined so that the lowest value of the "high *AHR*" category was much higher than the highest value of the "low *AHR*" category. We chose a threshold of significance of p<0.01 to not find genes differentially expressed by chance, which can happen when a large number of variables are studied. *AHR* mRNA expression was significantly different between the low *AHR*-expressing and high *AHR*-expressing breast tumor groups in both the ERα-negative (p<0.0000001) and ERα-positive (p = 0.0000067) subpopulations (Table 4).

**Table 4:** Statistical analysis of mRNA expression of AHR signaling pathway genes relative to *AHR* mRNA expression and ERα status in two breast tumor subpopulations.

Genes	ERα-negative breast tumor subpopulation			ERα-positive breast tumor subpopulation		
	Low *AHR* mRNA expression group (n = 26)	High *AHR* mRNA expression group (n = 26)	*p*-value^a^	Low *AHR* mRNA expression group (n = 14)	High *AHR* mRNA expression group (n = 14)	*p*-value^a^
Control genes (n = 3)						
***AHR***	26.0 (2.97–49.3)^b^	81.3 (57.8–331)	**<0.0000001**	20.7 (0.20–56.6)	159 (74.4–310)	**0.0000067**
***AHRR***	4.10 (0.67–60.9)	9.62 (0.44–39.3)	**0.0036**	4.20 (0.0–9.71)	4.20 (3.15–35.2)	NS
***ARNT***	23.4 (8.32–56.7)	49.3 (20.8–151)	**0.0000042**	28.4 (4.30–68.0)	55.3 (24.1–92.5)	**0.0052**
Nuclear receptors (n = 6)						
***ESR1***	201 (12.5–1882)	238 (12.5–1819)	NS	15055 (3375–34878)	13550 (3300–39645)	NS
***ESR2***	1.44 (0.0–6.43)	2.47 (0.0–19.8)	0.018	0.96 (0.0–2.58)	0.80 (0.0–12.5)	NS
***AR***	562 (0.0–6400)	192 (0.0–6053)	NS	2220 (1103–4588)	1754 (940–3404)	NS
***PR***	6.71 (0.84–308)	8.39 (0.84–126)	NS	320 (5.03–4009)	252 (3.35–1124)	NS
***ESRRA***	112 (41.7–433)	192 (64.4–1223)	0.018	49.2 (22.3–191)	84.4 (23.2–260)	NS
***ESRRG***	13.3 (1.02–232)	9.35 (0.0–171)	NS	9.21 (0.42–124)	42.2 (4.36–133)	NS
Angiogenesis (n = 1)						
***VEGFA***	701 (196–2265)	1122 (268–6621)	NS	319 (135–919)	486 (187–1970)	NS
Cell proliferation (n = 4)						
***KI67***	927 (46.8–4055)	1184 (36.5–3497)	NS	413 (140–1038)	468 (99.6–1519)	NS
***CDKN2A***	1.14 (0.0–51.8)	2.04 (0.0–52.8)	NS	0.06 (0.0–1.56)	0.48 (0.0–2.96)	NS
***CDKN2B***	80.1 (9.45–475)	118 (26.7–1369)	NS	29.3 (0.0–174)	76.5 (13.6–169)	NS
***CCND1***	746 (98.8–3756)	1256 (180–8763)	NS	1244 (647–7427)	1510 (608–3342)	NS
EMT (n = 4)						
***VIM***	19421 (1494–42826)	22409 (7470–107563)	NS	16931 (6972–41830)	21413 (8964–41830)	NS
***CDH1***	5244 (519–14866)	7779 (57.6–31980)	NS	6742 (3457–35667)	6108 (1210–35494)	NS
***SNAI1***	35.5 (9.86–226)	60.4 (11.7–493)	0.018	16.8 (6.34–58.4)	31.1 (8.67–72.7)	NS
***SNAI2***	202 (56.7–764)	207 (76.3–1843)	NS	137 (48.6–394)	236 (80.1–389)	NS
Cell motility (n = 6)						
***MMP1***	19.1 (0.50–266)	97.3 (0.50–1055)	**0.0024**	3.47 (0.0–5.94)	12.8 (0.0–544)	NS
***MMP2***	676 (88.7–2105)	1364 (288–9906)	**0.0022**	646 (125–1917)	1332 (370–2855)	0.021
***MMP9***	270 (5.21–885)	410 (75.3–10072)	0.04	86.4 (23.2–455)	218 (53.6–749)	0.031
***MMP13***	42.4 (0.0–410)	67.8 (0.0–1367)	NS	26.6 (0.99–131)	60.4 (0.0–434)	NS
***MMP14***	1064 (178–3396)	1291 (573–20614)	0.041	503 (24.7–1446)	1167 (2.97–2900)	0.022
***PLAU***	216 (54.1–810)	391 (143–4171)	**0.0032**	140 (43.7–1154)	357 (87.1–890)	0.022
IGF pathway (n = 5)						
***IGF1R***	306 (81.3–1492)	312 (85.3–1824)	NS	736 (259–2043)	1568 (584–3488)	**0.0088**
***IGF2***	565 (42.4–5243)	515 (70.6–53691)	NS	915 (164–3563)	1054 (425–6293)	NS
***IGF2R***	935 (505–2227)	1174 (263–2951)	**0.0082**	525 (305–863)	1003 (420–1711)	**0.00044**
***IGFBP5***	2517 (302–23846)	2835 (427–15431)	NS	5800 (988–266078)	3175 (490–26137)	NS
***TGFB1***	666 (114–1169)	738 (275–3982)	NS	451 (228–800)	835 (269–2014)	**0.0035**
Inflammation (n = 10)						
***IL1B***	9.07 (1.86–49.5)	29.4 (6.40–116)	**0.000013**	9.36 (1.58–66.4)	15.8 (1.06–42.2)	NS
***IL6***	1.39 (0.13–12.5)	3.77 (0.81–132)	**0.0013**	0.34 (0.0–13.9)	1.90 (0.31–14.9)	**0.0033**
***TNF***	48.5 (14.4–234)	98.9 (19.7–524)	**0.008**	43.5 (13.0–136)	49.5 (16.3–152)	NS
***IL8***	145 (22.0–2068)	492 (9.25–8412)	**0.00087**	39.0 (9.24–177)	41.4 (8.34–566)	NS
***PTGS2***	18.4 (2.71–3373)	30.3 (3.68–1643)	0.038	12.1 (1.60–57.3)	8.18 (4.44–55.9)	NS
***CSF1***	308 (61.7–1273)	455 (145–1759)	0.039	309 (102–739)	561 (296–2173)	**0.0051**
***CSF1R***	481 (112–1382)	825 (181–8703)	0.013	401 (222–1071)	860 (209–1132)	NS
***CXCR4***	1.54 (0.0–40.0)	4.47 (0.0–30.9)	**0.0035**	0.56 (0.0–10.2)	1.96 (0.26–10.3)	NS
***CXCL12***	1664 (83.0–4508)	1823 (470–9587)	NS	1369 (355–5345)	1550 (725–5653)	NS
***CCL5***	417 (81.0–5016)	723 (200–7161)	NS	288 (64.6–1198)	240 (85.7–1382)	NS
Chromatin structure regulation (n = 5)						
***HOTAIR***	42.3 (0.0–261)	20.2 (0.0–221)	NS	4.69 (0.0–104)	13.4 (0.0–89.4)	NS
***ANRIL***	24.6 (0.66–106)	30.4 (7.61–124)	NS	18.2 (8.1–76.7)	19.4 (5.07–56.5)	NS
***EZH2***	229 (23.7–1345)	241 (30.7–488)	NS	86.3 (51.6–454)	99.9 (27.0–260)	NS
***SUZ12***	480 (193–2790)	582 (222–1409)	NS	615 (234–19545)	516 (393–685)	NS
***CBX7***	64.1 (12.7–280)	76.8 (18.9–486)	NS	80.0 (30.7–281)	83.5 (34.5–212)	NS
DNA repair (n = 1)						
***BRCA1***	0.40 (0.0–2.02)	1.84 (0.0–10.1)	**0.0000023**	0.47 (0.0–4.18)	1.97 (0.55–12.4)	0.012
Upstream signaling (n = 3)						
***IDO1***	2.85 (0.0–106)	15.8 (0.85–250)	**0.0041**	0.60 (0.0–10.0)	2.30 (0.42–11.2)	0.015
***IDO2***	0.06 (0.0–4.05)	0.99 (0.0–10.9)	NS	0.0 (0.0–1.41)	0.36 (0.0–1.31)	NS
***TDO2***	7.47 (0.74–96.6)	22.6 (1.19–101)	**0.0015**	1.76 (0.0–21.8)	4.89 (0.39–42.7)	NS
Xenobiotic metabolism and/or cholesterol synthesis (n = 8)						
***CYP1A1***	0.0 (0.0–5.09)	0.0 (0.0–1.60)	NS	0.0 (0.0–0.21)	0.0 (0.0–5.03)	NS
***CYP1A2***	NE	NE	-	NE	NE	-
***CYP1B1***	199 (43.2–5864)	478 (23.4–5921)	0.027	161 (22.8–344)	163 (28.5–427)	NS
***CYP2B6***	2.12 (0.0–204)	4.33 (0.0–2426)	NS	234 (19.8–4047)	1878 (6.13–27916)	NS
***CYP4B1***	9.87 (0.0–1409)	14.6 (0.0–147)	NS	310 (18.1–584)	178 (3.24–1934)	NS
***CYP4X1***	87.4 (1.08–2427)	55.1 (0.0–5112)	NS	218 (6.84–5632)	1095 (1.93–4997)	NS
***NOS2A***	0.97 (0.23–8.38)	1.31 (0.0–5.83)	NS	0.38 (0.0–5.02)	1.40 (0.0–3.88)	NS
***NQO1***	438 (31.2–1808)	342 (49.2–2988)	NS	577 (118–2995)	481 (223–2778)	NS

Abbreviations: ERα: estrogen receptor alpha; NS: not significant; NE: not expressed.

Values shown in bold type are statistically significant at confidence levels greater than 99% (*p*-value<0.01).

^a^ Kruskal-Wallis H Test.

^b^ Median (range) of gene mRNA levels; mRNA expression level relative to Ct 35 = 1.

*ARNT* mRNA levels were also increased in the highest *AHR*-expressing groups independently of ERα status. *AHRR* mRNA level was significantly twofold higher in high *AHR*-expressing breast tumors compared to low *AHR*-expressing tumors, but only in the ERα-negative subpopulation (p = 0.0036) (Table 4). Little or no correlations were observed between *AHR* mRNA expression level and nuclear receptors including ERα, PR and AR (Table 4).

In addition to *ARNT*, *AHRR* and genes encoding nuclear receptors (n = 6), genes reported in the literature to be involved in *AHR* signaling pathways, and various well known cancer pathways, including angiogenesis (n = 1), cell proliferation (n = 4), epithelial-mesenchymal transition (EMT) (n = 4), cell motility (n = 6), IGF pathway (n = 5), inflammation (n = 10), chromatin structure regulation (n = 5), DNA repair (n = 1), and upstream and downstream metabolism of AHR signaling (n = 3 and n = 8, respectively) were also analyzed (Table 4). Little or no correlations were observed between *AHR* mRNA expression level and angiogenesis, cell proliferation, EMT, chromatin structure and, surprisingly, xenobiotic metabolism gene expression levels, which are well known AHR-inducible genes (Table 4). More interestingly, *MMP1*, *MMP2* and *PLAU* mRNA expressions were significantly associated with the high *AHR*-expressing group in the ERα-negative subpopulation (p = 0.0024, 0.0022 and 0.0032, respectively) (Table 4). A weaker correlation was also observed between *AHR* mRNA levels and *MMP9* and *MMP14*. These results suggest that AHR plays an important role in cell motility, mainly in ERα-negative breast tumors. Among the five genes selected from the IGF pathway, *IGF2R* expression was significantly increased in the highest *AHR*-expressing groups, independently of ERα status (p = 0.00044 in ERα-positive tumors and p = 0.0082 in ERα-negative tumors, respectively) (Table 4). *IGF1R* and *TGFB1* were significantly overexpressed in the highest *AHR*-expressing group, but only in ERα-positive breast tumors (p = 0.0088 and p = 0.0035, respectively) (Table 4).

*IL1B*, *IL6*, *TNF*, *IL8* and *CXCR4*, involved in the inflammation pathway, were significantly overexpressed in the high *AHR*-expressing group compared to the low *AHR*-expressing group in the ERα-negative subpopulation (Table 4). Furthermore, these associations were not observed in the ERα-positive subgroup, except for *IL6* and *CSF1*. The majority of these positive correlations (in particular for *IL1B* and *IL6*, *p*-value<0.01) were confirmed in the TCGA breast cancer dataset (data not shown) [45]. Higher *AHR* mRNA expression levels therefore appear to be strongly involved in inflammation processes, mainly in ERα-negative breast tumors.

Other genes that have recently been shown to be involved in tryptophan metabolism via the kynurenine pathway were also analyzed [46]. *IDO1* mRNA levels were significantly increased in high *AHR*-expressing breast tumors relative to low *AHR*-expressing breast tumors in both ERα subpopulations (Table 4). *TDO2* mRNA levels were also significantly increased in high *AHR*-expressing breast tumors, but only in the ERα-negative subpopulation (Table 4). These positive correlations, in particular for *IDO1* (*p*-value<0.01), were also confirmed with the data of The Cancer Genome Atlas Breast invasive carcinoma project (data not shown) [45].

Finally, *BRCA1* mRNA expression was strongly associated with high *AHR*-expressing breast tumors in the ERα-negative subpopulation (p = 0.0000023) (Table 4). A similar, approximately fourfold difference in *BRCA1* mRNA expression was observed between the high and low *AHR* mRNA expression groups in both ERα-positive and ERα-negative subpopulations. However, a weaker association was observed in the ERα-positive subpopulation (p = 0.012) compared to the ERα-negative subpopulation.

### Effect of two different *AHR* ligands on mRNA expressions of *AHR*, *AHRR* and *ARNT*, and of genes involved in inflammation, in MDA-MB-436 breast cancer cell line

**Fig 2:**
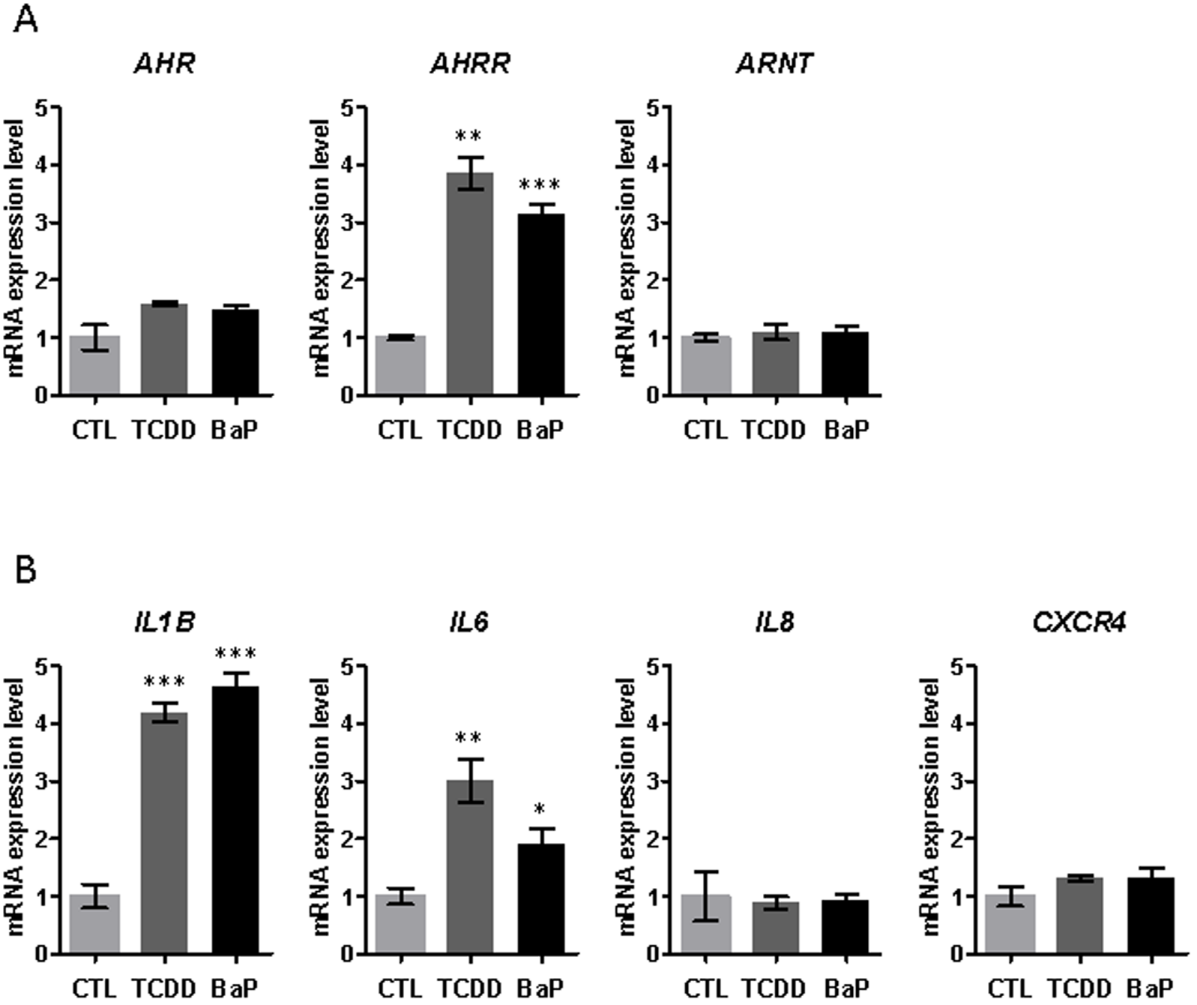
mRNA expression levels of *AHR*, *AHRR* and *ARNT*, and of genes involved in inflammation, in MDA-MB-436 breast cancer cell line treated with two different *AHR* ligands. MDA-MB-436 cells were cultivated in absence (CTL) or in presence of TCDD (2,3,7,8-tetrachlorodibenzo-p-dioxin) (10^-9^M) or BaP (benzo[a]pyrene) (10^-6^M) for 16 h. Cells were then lysed and mRNA extracted. mRNA expression levels of *AHR*, *AHRR* and *ARNT* (A), and of *IL1B*, *IL6*, *IL8* and *CXCR4* (B) were determined by qRT-PCR. All experiments were performed in triplicate. Results were expressed as mean +/- s.e.m and normalized so that the mean of the control cells was 1. Three levels of statistical significance are distinguished: *p-value<0.05; **p-value<0.01; ***p-value<0.001.

In order to confirm the implication of *AHR* in the regulation of inflammation genes, we examined the effect of two *AHR* ligands: TCDD (2,3,7,8-tetrachlorodibenzo-p-dioxin) and BaP (benzo[a]pyrene), on mRNA expression of several inflammation genes: *IL1B*, *IL6*, *TNF*, *IL8* and *CXCR4*, in MDA-MB-436 ERα-negative breast cancer cells. First, to test the activity of the two ligands, we determined their effects on the expression of *AHR*, *AHRR* and *ARNT*. We did not detect significant effect of the ligands on *AHR* and *ARNT* expressions. However, the ligands strongly stimulated the expression of *AHRR* (*AHR* repressor). The two ligands induced therefore a negative feedback loop, indicating that they are active in our experimental conditions [24]. Moreover, we found that mRNA expression levels of *IL1B* and *IL6* were significantly higher in cells treated with TCDD or BaP compared to control cells (Fig 2) confirming the positive effect of *AHR* on the regulation of inflammation genes. We were not able to study the effect of our ligands on the expressions of *TNF* and of genes of the endogenous tryptophan metabolism pathway (*IDO1*, *IDO2* and *TDO2*), because of the absence of expression of these genes in this cell line.

### AhR protein is present in breast cancer tissues

**Fig 3:**
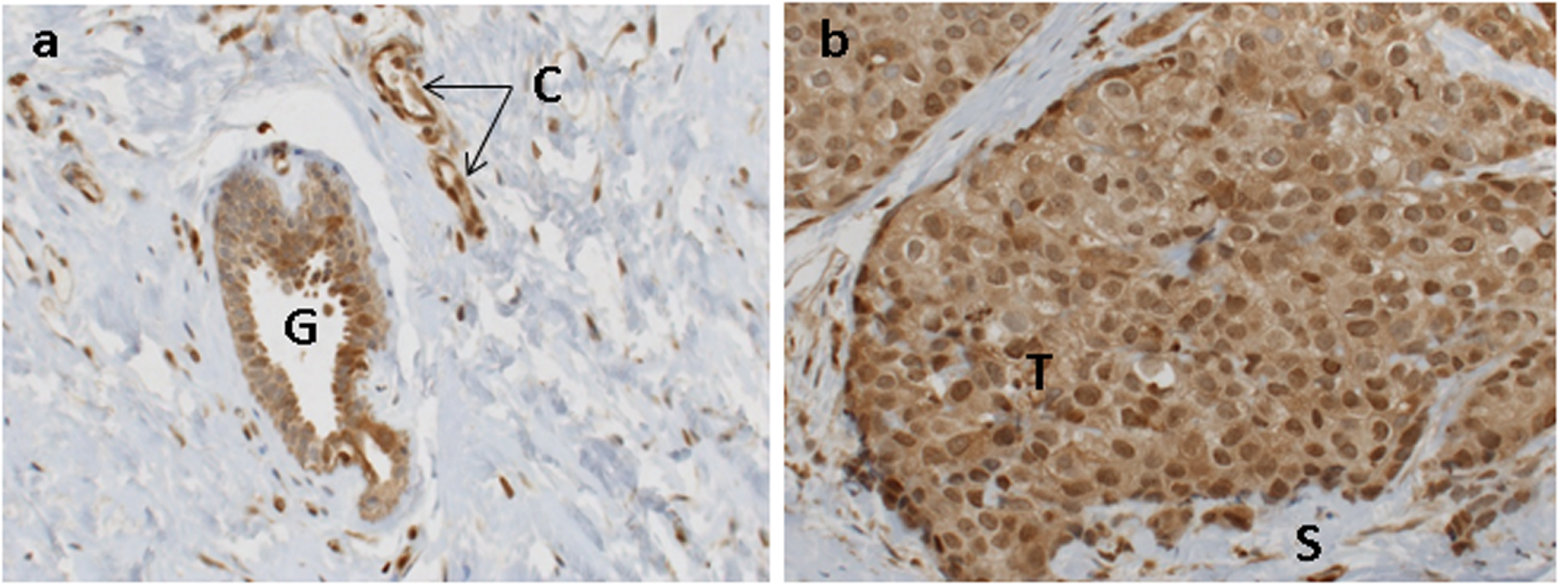
Immunocytochemical staining for AhR in human breast tissue. a, peritumoral “normal” tissue. b, tumor tissue. Note the intense staining in both nuclei and cytoplasm in b. G, epithelial glands; C, capillaries; T, tumor cells; S, intratumoral stroma. Original Magnification, x 20.

**Fig 4:**
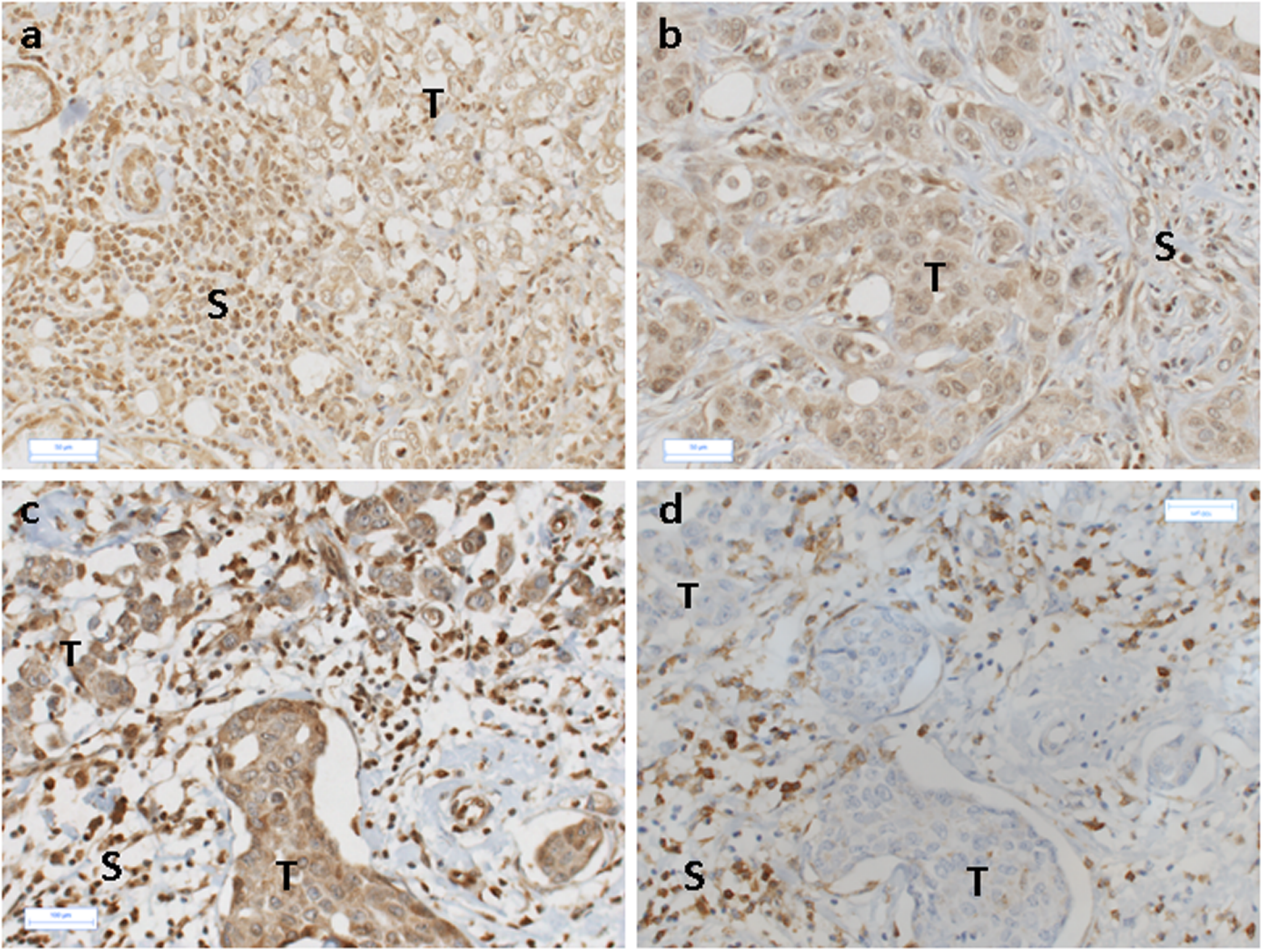
Immunocytochemical staining for AhR and CD4 in breast tumors. **a-b**, moderate AhR-expressing tumor cells. Note positive AhR staining in the intratumoral stroma. **c-d**, immunostaining for AhR (c) or CD4 (d) in the same tumor sample. Immunostaining for both AhR and CD4 is observed in stromal cells in the intratumoral compartment. T, tumor cells; S, intratumoral stroma. Original Magnification, x 20.

In line with the objective of this study, immunohistochemistry was performed on paraffin sections to assess the localization of AhR protein on a total of 30 ERα-positive or ERα-negative breast tumors. These tumors corresponded to a panel of freshly excised tumors ranging from grade 1 to grade 3. AhR immunostaining in peritumoral tissue ("normal" tissue adjacent to the tumor) was mainly observed in epithelial (glandular) cells and capillaries (Fig 3a). Tumor cells and intratumoral stroma were immunostained for AhR (Fig 3b). The sub-localization of AhR (nuclear or cytoplasmic) was analyzed in tumor cells from all samples. 100% of tumor samples were positively stained for cytoplasmic AhR in breast tumor cells. However, AhR immunostaining was also observed in both the cytoplasm and nuclei in 24/30 tumors (80%) (Fig 3b). The intensity of AhR immunostaining in tumor cells varied from strong (Fig 3b) to low or moderate (Fig 4a–4c), depending on the individual tumor. AhR immunostaining was also observed in both nuclei and cytoplasm of tumor cells (Fig 3b). In addition, AhR was present in the intratumoral nonepithelial tissue (Figs 3b and 4a), including endothelial cells and immune cells including lymphocytes. The presence of AhR in tumor-infiltrating lymphocytes was confirmed by the use of CD4 antibodies (Fig 4d). The expression of Cyp1B1 protein, a known target of activated AhR, was also analyzed in tumors with high or low AhR protein levels. No correlation was observed between Cyp1B1 and high (or low) AhR protein levels in epithelial cells (data not shown), thereby confirming the mRNA expression results.

## Discussion

This study suggests that the level of *AHR* expression could play an important role in breast tumorigenesis. Tumors with high levels of *AHR* show increased expression of genes involved in several pathways, including invasion, IGF signaling, inflammation, DNA repair and kynurenine metabolism. ER status influences the correlation between *AHR* expression and the transcription of most of these genes in breast cancers. We also show, for the first time, that *AHRR* status is associated with MFS and is an independent prognostic factor, while *AHR* is not an independent prognostic factor. In this study, the gene expression level was assessed using a quantitative real-time RT-PCR method. This single-step method has several advantages as compared to microarrays or RNAseq, including higher accuracy in the quantification procedure, and higher sensitivity.

### Expression of *AHR*, *AHRR* and *ARNT*

In a series of 439 ERα-positive or ERα-negative breast tumors, representing the different molecular subtypes, large ranges of *AHR* (0–5.8-fold difference) and *AHRR* (0–19.8-fold difference) mRNA expression were observed, reflecting extensive heterogeneity of gene expression in tumor samples. When comparing *AHR* and *AHRR*, *AHR* was associated with age, ER and HR negative status, and HR-/ERRB2- subtype, whereas *AHRR* was independent of age, ER and HR negative status, and HR-/ERRB2- subtype. Higher basal *AHR* expression in triple-negative breast cancer has been previously reported [47–49]. The present study did not reveal any correlation between *AHR* and *MKI67* or *EGFR* mRNA levels, or between *AHR* and PIK3CA mutation status, suggesting that high *AHR mRNA* levels are not implicated in cell proliferative activity. The strong correlation between high *AHR* and *ARNT* levels in both ERα-negative and ERα-positive tumors (Table 4) also suggests that the heterodimer, AHR-ARNT (AHR nuclear translocator), could be active in breast tumors.

The immunocytochemical results indicated a role for AhR in both tumor cells and the tumor microenvironment. Saito (2014) previously reported that AhR status was inversely correlated with the histological grade of invasive ductal carcinoma [38]. However, in their study, AhR status was based on a 10% immunocytochemical positivity threshold of carcinoma cells in breast tumors (n = 90) [38], while the present study analyzed both on AhR protein and mRNA. AhR protein was observed in both tumor cells and the tumor microenvironment (intratumoral stroma). AhR immunostaining was observed in the cytoplasm of tumor cells in the 30 tumors tested, and nuclear immunostaining was also observed in 24/30 tumors (80%). Nuclear AhR localization suggests AhR activation in breast cancers due to the presence of exogenous or endogenous ligands.

*AHR* mRNA levels in breast cancers were not a prognostic factor for patient survival, as also reported for colon, pancreas, stomach and thyroid cancers, which express high *AHR* mRNA levels [44]. Notably, our results revealed, for the first time, that breast tumors with high *AHRR* mRNA levels were significantly associated with good metastasis-free survival, compared to tumors expressing low *AHRR* mRNA levels. These results raise the novel suggestion that *AHRR* levels may represent an independent prognostic factor for breast cancer. This correlation has been previously reported for other tumors, including colon, lung, stomach and ovarian cancer [43]. *AHRR* acts as a tumor suppressor gene in several types of cancer cells. Knock-out of *AHRR* in mammary epithelial cells enables them to grow in an anchorage-dependent manner [43]. AhRR is an evolutionarily conserved bHLH-PAS protein that inhibits both xenobiotic-induced and constitutively active *AHR* transcriptional activity in multiple species including humans [23–24]. AhRR functions as a feedback modulator by competing with the transcription factor for heterodimer formation with ARNT [23–24]. *AHRR* expression is induced by the AhR/ARNT heterodimer *via* binding to xenobiotic response elements (XREs) located in the 5’ flanking region of the *AHRR* gene. Unfortunately, AhRR protein levels could not be assessed due to the lack of suitable antibodies.

To identify genes that may be correlated with *AHR* levels and AhR signaling pathways in breast cancers, 54 candidate genes were selected from the two ERα subpopulations expressing high or low *AHR* levels. These genes were chosen on the basis of data of the literature for their involvement in AHR signaling pathways including cell motility, IGF pathway, inflammation, DNA repair, and upstream and downstream metabolism.

### High *AHR* expression correlates with expression of genes involved in inflammation

*IL1B*, *IL6*, *TNF*, *IL8* and *CXCR4* mRNA levels were significantly increased in the high *AHR*-expressing ERα-negative breast tumor subpopulation, while these associations were only observed for *IL6* and *CSF1* in the ERα-positive tumor subgroup. Moreover, mRNA expression levels of *IL1B* and *IL6* were significantly higher in MDA-MB-436 cells treated with TCDD or BaP compared to control cells confirming the positive effect of *AHR* on the regulation of inflammation genes. These results confirm and extend *in vitro* results showing that AhR activation promotes induction of *IL6* [49–51]. Gene expression of *IL6*, a cytokine involved in immune cell homeostasis that elicits protumor and antitumor properties, has also been shown to be synergistically induced by stimulation of AhR activity in combination with IL1B or TNF in MCF-7 cells [50]. These results further confirm the important role of AhR in the regulation of inflammation.

### High *AHR* expression correlates with several metalloproteases and genes involved in IGF signaling

Several proteases, including MMP1, MMP2, MMP9, MMP14 and u-PA, are involved in invasive breast tumor growth and metastasis [52]. Interestingly, high levels of *MMP2* and *PLAU* that encodes uPA mRNAs were shown to be significantly associated with high *AHR* expression in addition to *MMP1* in ERα-negative tumors. Other *MMPs* such as *MMP9* and *MMP14* were more weakly associated with *AHR* expression. These results also suggest an important role of *AHR* expression in breast tumor cell motility. A positive correlation was also demonstrated between *AHR* expression and *IGF2R* expression. In contrast, *IGF1R* expression was correlated with high *AHR* mRNA expression only in ERα-positive breast tumors, as previously described [53]. These results add support to the involvement of AHR, together with IGF1R and IGF2R, in the IGF signaling pathway, depending on the breast tumor group. Another novel finding of this study is that *BRCA1* expression is strongly associated with the high *AHR*-expressing ERα-negative subpopulation, indicating a possible implication of *AHR* in DNA repair in ERα-negative breast cancers.

### High AhR expression correlates with expression of genes involved in tryptophan metabolism

The levels of expression of *IDO1*, *IDO2* and *TDO2*, involved in the early steps of tryptophan metabolism leading to kynurenine, an AhR ligand were analyzed [12,46]. Interestingly, *IDO1* levels, but not *IDO2 levels* (not expressed), were significantly elevated in high *AHR*-expressing breast tumors compared to low *AHR*-expressing breast tumors in both ERα subpopulations. The IDO1 enzyme (indoleamine-2,3-dioxygenase) mediates the first rate-limiting step by converting tryptophan metabolites into L-kynurenine and is upregulated in an inflammatory microenvironment (e.g. in the presence of IL6) [54]. IDO1 enzyme activity may lead to a local “amino-acid starvation” response. By generating downstream metabolites, IDO1 enzyme activity may also affect immunity, including specific immunomodulatory or cytotoxic functions [55]. Recent studies have shown that tryptophan metabolites can alter the balance of Treg and Th17, two related populations of CD4+ T cells with opposing functions during immune responses [32]. *IDO1* expression and differentiation of the common precursor of these immune cells may be governed by the presence of inflammatory cytokines [16]. L-kynurenine has also been reported to activate AhR [5], which positively regulates *IDO1* expression by immune cells such as dendritic cells. *TDO2* mRNA levels were also significantly increased in high *AHR*-expressing breast tumors, but only in the ERα-negative subpopulation. A strong correlation was therefore demonstrated between *AHR*, *IDO1* and *TDO2* expression in breast tumors. Whether the level of *AHR* is mainly due to kynurenine involving an autocrine loop, or due to the presence of an exogenous ligand has yet to be elucidated.

ER status is likely to influence the correlation between *AHR* expression and transcription of most of these genes in breast cancers. Following ligand binding in breast cancer cells, AhR can activate two pathways, an X/DRE-mediated DNA binding pathway and/or a non-X/DRE-mediated protein-protein interaction pathway, both of which can lead to changes in gene expression. The DRE-mediated pathway is the classical AhR pathway that leads to induction of dioxin-dependent genes, such as *CYP1A1* and *CYP1B1* and other genes including *IL1B*, *IL6* and *AHRR* [10,56], as also observed in the present work, via direct binding of the AhR complex to the promoter containing a DRE (an AhR/ARN-T binding motif). In addition to this canonical AhR pathway, alternative pathways for AhR-mediated transcriptional regulation have also been described, in which ligand-bound AhR interacts with other signaling pathways (ERα, SP1 and NF-KB) to regulate gene transcription. This process involves protein-protein interaction. Many studies have suggested cross-talk between AhR and ERα in ERα-positive breast cancer cells [25,57–59]. *TGFB1*, *TNF*, *IL1B* and *IL6* are inhibited by ligand-bound AhR in ERα-positive breast cancer cells. Cross-talk between AhR and NF-KB pathways has also been implicated in the regulation of AhR-mediated gene transcription such as *IL6* and *IL8* in breast cancers [60–61].

Our findings documenting *AhR* expression levels may contribute to targeting AhR for breast cancer therapy. A complete overview of the role of AhR in breast tumor growth is not currently available, especially as most studies have been conducted on cell culture and rodent models. AhR plays a key role in driving normal mammary gland development, and in driving breast cancer progression [4,28]. AhR influences the major stages of tumorigenesis: initiation, promotion, progression and metastasis. Various classes of AhR ligands may influence tumorigenic outcome, especially in aggressive breast tumors [37,48,62–65]. A major gap in our understanding of AhR activity in mammary tumors is the nature of the signals that drive AhR activation. In particular, the contribution of endogenous ligands *vs* exogenous ligands in various breast tumor types remains unknown. The presence of high-affinity AhR ligands can produce substantial AhR transcriptional activity, even in the presence of modest levels of AhR expression. The relative expression of AhR may be a determinant factor in the presence of low-affinity ligands. In the present study, none of the genes involved in xenobiotic metabolism (such as *CYP1B1*, the most extensively studied AhR-activated gene) were significantly increased with high AhR levels.

In conclusion, the results reported here represent progress compared to previous studies that focused on breast cancer cells as models for determining the mechanisms and pathways activated by TCDD, the main potent AhR ligand. The role of AhR in breast tumorigenesis has also been demonstrated in animal models. The present study documents the variable expression of *AhR* in tumors, both in tumor cells and in other cells present in the tumor microenvironment, confirming the complexity of AhR functions in breast cancer. Several genes whose expression is correlated with *AHR* expression and which are involved in various signaling pathways related to AhR activation were identified in this study. A major gap in our understanding of AhR activity in mammary tumors concerns the nature (exogenous or endogenous) of the signal that constitutively drives AhR activation. The present results suggest that *AHR* expression levels could also be discriminant in patients with hormone receptor positive and negative breast cancers and also indicate that AhR is a target for breast cancer therapy. The role of AhR in breast cancers, in the presence of endogenous or exogenous AhR ligands, merits further examination, by distinguishing ERα-positive from ERα-negative breast cancers.

## References

[1] Madigan MP, Ziegler RG, Benichou J, Byrne C, Hoover RN. Proportion of breast cancer cases in the United States explained by well-established risk factors. J Natl Cancer Inst. 1995;87(22):1681–1685. 7473816 10.1093/jnci/87.22.1681

[2] Lichtenstein P, Holm NV, Verkasalo PK, Iliadou A, Kaprio J, Koskenvuo M et al Environmental and heritable factors in the causation of cancer—analyses of cohorts of twins from Sweden, Denmark, and Finland. N Engl J Med. 2000;343(2):78–85. doi: 10.1056/NEJM200007133430201 10891514 10.1056/NEJM200007133430201

[3] Birnbaum LS, Fenton SE. Cancer and developmental exposure to endocrine disruptors. Environ Health Perspect. 2003;111(4):389–394. 12676588 10.1289/ehp.5686 PMC1241417

[4] Fenton SE, Reed C, Newbold RR. Perinatal environmental exposures affect mammary development, function, and cancer risk in adulthood. Annu Rev Pharmacol Toxicol. 2012;52:455–479. doi: 10.1146/annurev-pharmtox-010611-134659 22017681 10.1146/annurev-pharmtox-010611-134659 PMC3477544

[5] Phrakonkham P, Brouland JP, Saad Hel S, Bergès R, Pimpie C, Pocard M et al Dietary exposure in utero and during lactation to a mixture of genistein and an anti-androgen fungicide in a rat mammary carcinogenesis model. Reprod Toxicol. 2015;54:101–109. doi: 10.1016/j.reprotox.2014.05.016 24915137 10.1016/j.reprotox.2014.05.016

[6] Brody JG, Moysich KB, Humblet O, Attfield KR, Beehler GP, Rudel RA. Environmental pollutants and breast cancer: epidemiologic studies. Cancer. 2007;109(12 Suppl):2667–2711. doi: 10.1002/cncr.22655 17503436 10.1002/cncr.22655

[7] Bertazzi PA, Zocchetti C, Guercilena S, Conconni D, Tironi A, Landi MT et al Dioxin exposure and cancer risk: a 15-year mortality study after the "Seveso accident". Epidemiology. 1997;8(6):646–652. 9345664

[8] Pesatori AC, Consonni D, Rubagotti M, Grillo P, Bertazzi PA. Cancer incidence in the population exposed to dioxin after the "Seveso accident": twenty years of follow-up. Environ Health. 2009;8:39 doi: 10.1186/1476-069X-8-39 19754930 10.1186/1476-069X-8-39 PMC2754980

[9] Warner M, Mocarelli P, Samuels S, Needham L, Brambilla P, Eskenazi B. Dioxin exposure and cancer risk in the Seveso Women’s Health Study. Environ Health Perspect. 2011;119(12):1700–1705. doi: 10.1289/ehp.1103720 21810551 10.1289/ehp.1103720 PMC3261987

[10] Denison MS, Nagy SR. Activation of the aryl hydrocarbon receptor by structurally diverse exogenous and endogenous chemicals. Annu Rev Pharmacol Toxicol. 2003;43:309–334. doi: 10.1146/annurev.pharmtox.43.100901.135828 12540743 10.1146/annurev.pharmtox.43.100901.135828

[11] Denison MS, Soshilov AA, He G, DeGroot DE, Zhao B. Exactly the same but different: promiscuity and diversity in the molecular mechanisms of action of the aryl hydrocarbon (dioxin) receptor. Toxicol Sci. 2011;124(1):1–22. doi: 10.1093/toxsci/kfr218 21908767 10.1093/toxsci/kfr218 PMC3196658

[12] Opitz CA, Litzenburger UM, Sahm F, Ott M, Tritschler I, Trump S et al An endogenous tumour-promoting ligand of the human aryl hydrocarbon receptor. Nature. 2011;478(7368):197–203. doi: 10.1038/nature10491 21976023 10.1038/nature10491

[13] Go RE, Hwang KA, Choi KC. Cytochrome P450 1 family and cancers. J Steroid Biochem Mol Biol. 2015;147:24–30. doi: 10.1016/j.jsbmb.2014.11.003 25448748 10.1016/j.jsbmb.2014.11.003

[14] Larsen MC, Angus WG, Brake PB, Eltom SE, Sukow KA, Jefcoate CR. Characterization of CYP1B1 and CYP1A1 expression in human mammary epithelial cells: role of the aryl hydrocarbon receptor in polycyclic aromatic hydrocarbon metabolism. Cancer Res. 1998;58(11):2366–2374. 9622076

[15] Barouki R, Coumoul X, Fernandez-Salguero PM. The aryl hydrocarbon receptor, more than a xenobiotic-interacting protein. FEBS Lett. 2007;581(19):3608–3615. doi: 10.1016/j.febslet.2007.03.046 17412325 10.1016/j.febslet.2007.03.046

[16] Roman AC, Carvajal-Gonzalez JM, Rico-Leo EM, Fernandez-Salguero PM. Dioxin receptor deficiency impairs angiogenesis by a mechanism involving VEGF-A depletion in the endothelium and transforming growth factor-beta overexpression in the stroma. J Biol Chem. 2009;284(37):25135–25148. doi: 10.1074/jbc.M109.013292 19617630 10.1074/jbc.M109.013292 PMC2757217

[17] Schlezinger JJ, Liu D, Farago M, Seldin DC, Belguise K, Sonenshein GE et al A role for the aryl hydrocarbon receptor in mammary gland tumorigenesis. Biol Chem. 2006;387(9):1175–1187. doi: 10.1515/BC.2006.145 16972784 10.1515/BC.2006.145

[18] Feng S, Cao Z, Wang X. Role of aryl hydrocarbon receptor in cancer. Biochim Biophys Acta. 2013;1836(2):197–210. doi: 10.1016/j.bbcan.2013.05.001 23711559 10.1016/j.bbcan.2013.05.001

[19] Murray IA, Patterson AD, Perdew GH. Aryl hydrocarbon receptor ligands in cancer: friend and foe. Nat Rev Cancer. 2014;14(12):801–814. doi: 10.1038/nrc3846 25568920 10.1038/nrc3846 PMC4401080

[20] Fernandez-Salguero P, Pineau T, Hilbert DM, McPhail, Lee SS, Kimura S et al Immune system impairment and hepatic fibrosis in mice lacking the dioxin-binding Ah receptor. Science. 1995;268(5211):722–726. 7732381 10.1126/science.7732381

[21] Stockinger B, Di Meglio P, Gialitakis M, Duarte JH. The aryl hydrocarbon receptor: multitasking in the immune system. Annu Rev Immunol. 2014;32:403–432. doi: 10.1146/annurev-immunol-032713-120245 24655296 10.1146/annurev-immunol-032713-120245

[22] Quintana FJ, Sherr DH. Aryl hydrocarbon receptor control of adaptive immunity. Pharmacol Rev. 2013;65(4):1148–1161. doi: 10.1124/pr.113.007823 23908379 10.1124/pr.113.007823 PMC3799235

[23] Mimura J, Ema M, Sogawa K, Fujii-Kuriyama Y. Identification of a novel mechanism of regulation of Ah (dioxin) receptor function. Genes Dev. 1999;13(1):20–25. 9887096 10.1101/gad.13.1.20 PMC316371

[24] Hahn ME, Allan LL, Sherr DH. Regulation of constitutive and inducible AHR signaling: complex interactions involving the AHR repressor. Biochem Pharmacol. 2009;77(4):485–497. doi: 10.1016/j.bcp.2008.09.016 18848529 10.1016/j.bcp.2008.09.016 PMC2701375

[25] Ohtake F, Takeyama K, Matsumoto T, Kitagawa H, Yamamoto Y, Nohara K et al Modulation of oestrogen receptor signalling by association with the activated dioxin receptor. Nature. 2003;423(6939):545–550. doi: 10.1038/nature01606 12774124 10.1038/nature01606

[26] Vogel CF, Li W, Wu D, Miller JK, Sweeney C, Lazennec G et al Interaction of aryl hydrocarbon receptor and NF-kappaB subunit RelB in breast cancer is associated with interleukin-8 overexpression. Arch Biochem Biophys. 2011;512(1):78–86. doi: 10.1016/j.abb.2011.05.011 21640702 10.1016/j.abb.2011.05.011 PMC3135412

[27] Guyot E, Chevallier A, Barouki R, Coumoul X. The AhR twist: ligand-dependent AhR signaling and pharmaco-toxicological implications. Drug Discov Today. 2013;18(9–10):479–486. doi: 10.1016/j.drudis.2012.11.014 23220635 10.1016/j.drudis.2012.11.014

[28] Brown NM, Manzolillo PA, Zhang JX, Wang J, Lamartiniere CA. Prenatal TCDD and predisposition to mammary cancer in the rat. Carcinogenesis. 1998;19(9):1623–1629. 9771934 10.1093/carcin/19.9.1623

[29] Kociba RJ, Keyes DG, Beyer JE, Carreon RM, Wade CE, Dittenber DA et al Results of a two-year chronic toxicity and oncogenicity study of 2,3,7,8-tetrachlorodibenzo-p-dioxin in rats. Toxicol Appl Pharmacol. 1978;46(2):279–303. 734660 10.1016/0041-008x(78)90075-3

[30] Knerr S, Schrenk D. Carcinogenicity of 2,3,7,8-tetrachlorodibenzo-p-dioxin in experimental models. Mol Nutr Food Res. 2006;50(10):897–907. doi: 10.1002/mnfr.200600006 16977593 10.1002/mnfr.200600006

[31] Brooks J, Eltom SE. Malignant transformation of mammary epithelial cells by ectopic overexpression of the aryl hydrocarbon receptor. Curr Cancer Drug Targets. 2011;11(5):654–669. 21486221 10.2174/156800911795655967 PMC4070443

[32] Wong PS, Li W, Vogel CF, Matsumura F. Characterization of MCF mammary epithelial cells overexpressing the Arylhydrocarbon receptor (AhR). BMC Cancer. 2009;9:234 doi: 10.1186/1471-2407-9-234 19604390 10.1186/1471-2407-9-234 PMC2721847

[33] Goode GD, Ballard BR, Manning HC, Freeman ML, Kang Y, Eltom SE. Knockdown of aberrantly upregulated aryl hydrocarbon receptor reduces tumor growth and metastasis of MDA-MB-231 human breast cancer cell line. Int J Cancer. 2013;133(12):2769–2780. doi: 10.1002/ijc.28297 23733406 10.1002/ijc.28297 PMC3797219

[34] Hall JM, Barhoover MA, Kazmin D, McDonnell DP, Greenlee WF, Thomas RS. Activation of the aryl-hydrocarbon receptor inhibits invasive and metastatic features of human breast cancer cells and promotes breast cancer cell differentiation. Mol Endocrinol. 2010;24(2):359–369. doi: 10.1210/me.2009-0346 20032195 10.1210/me.2009-0346 PMC2817602

[35] Mulero-Navarro S, Pozo-Guisado E, Perez-Mancera PA, Alvarez-Barrientos A, Catalina-Fernandez I, Hermandez-Nieto E et al Immortalized mouse mammary fibroblasts lacking dioxin receptor have impaired tumorigenicity in a subcutaneous mouse xenograft model. J Biol Chem. 2005;280(31):28731–28741. doi: 10.1074/jbc.M504538200 15946950 10.1074/jbc.M504538200

[36] Dohr O, Vogel C, Abel J. Different response of 2,3,7,8-tetrachlorodibenzo-p-dioxin (TCDD)-sensitive genes in human breast cancer MCF-7 and MDA-MB 231 cells. Arch Biochem Biophys. 1995;321(2):405–412. doi: 10.1006/abbi.1995.1411 7646066 10.1006/abbi.1995.1411

[37] D’Amato NC, Rogers TJ, Gordon MA, Greene LI, Cochrane DR, Spoelstra NS et al A TDO2-AhR signaling axis facilitates anoikis resistance and metastasis in triple-negative breast cancer. Cancer Res. 2015;75(21):4651–4664. doi: 10.1158/0008-5472.CAN-15-2011 26363006 10.1158/0008-5472.CAN-15-2011 PMC4631670

[38] Saito R, Miki Y, Hata S, Takagi K, Lida S, Oba Y et al Aryl hydrocarbon receptor in breast cancer-a newly defined prognostic marker. Horm Cancer. 2014;5(1):11–21. doi: 10.1007/s12672-013-0160-z 24078229 10.1007/s12672-013-0160-z PMC10358065

[39] Puccetti P, Fallarino F, Italiano A, Soubeyran I, MacGrogan G, Debled M et al Accumulation of an endogenous tryptophan-derived metabolite in colorectal and breast cancers. PLoS One. 2015;10(4):e0122046 doi: 10.1371/journal.pone.0122046 25881064 10.1371/journal.pone.0122046 PMC4400104

[40] Perrot-Applanat M, Groyer-Picard MT, Lorenzo F, Jolivet A, Vu Hai MT, Pallud C et al Immunocytochemical study with monoclonal antibodies to progesterone receptor in human breast tumors. Cancer Res. 1987;47(10):2652–2661. 2436753

[41] Hanley JA, McNeil BJ. The meaning and use of the area under a receiver operating characteristic (ROC) curve. Radiology. 1982;143(1):29–36. doi: 10.1148/radiology.143.1.7063747 7063747 10.1148/radiology.143.1.7063747

[42] Cox DR. Regression Models and Life-Tables. J R Stat Soc Series B Methodol. 1972;34(2):187–220.

[43] Zudaire E, Cuesta N, Murty V, Woodson K, Adams L, Gonzalez N et al The aryl hydrocarbon receptor repressor is a putative tumor suppressor gene in multiple human cancers. J Clin Invest. 2008;118(2):640–650. doi: 10.1172/JCI30024 18172554 10.1172/JCI30024 PMC2157559

[44] Safe S, Lee SO, Jin UH. Role of the aryl hydrocarbon receptor in carcinogenesis and potential as a drug target. Toxicol Sci. 2013;135(1):1–16. doi: 10.1093/toxsci/kft128 23771949 10.1093/toxsci/kft128 PMC3748760

[45] Cancer Genome Atlas Network. Comprehensive molecular portraits of human breast tumors. Nature. 2012;490:61–70. doi: 10.1038/nature11412 23000897 10.1038/nature11412 PMC3465532

[46] Mezrich JD, Fechner JH, Zhang X, Johnson BP, Burlingham WJ, Bradfield CA. An interaction between kynurenine and the aryl hydrocarbon receptor can generate regulatory T cells. J Immunol. 2010;185(6):3190–3198. doi: 10.4049/jimmunol.0903670 20720200 10.4049/jimmunol.0903670 PMC2952546

[47] Romagnolo DF, Papoutsis AJ, Laukaitis C, Selmin OI. Constitutive expression of AhR and BRCA-1 promoter CpG hypermethylation as biomarkers of ERalpha-negative breast tumorigenesis. BMC Cancer. 2015;15:1026 doi: 10.1186/s12885-015-2044-9 26715507 10.1186/s12885-015-2044-9 PMC4696163

[48] Powell JB, Goode GD, Eltom SE. The Aryl Hydrocarbon Receptor: A Target for Breast Cancer Therapy. J Cancer Ther. 2013;4(7):1177–1186. doi: 10.4236/jct.2013.47137 25068070 10.4236/jct.2013.47137 PMC4111475

[49] Salisbury TB, Tomblin JK, Primerano DA, Boskovic G, Fan J, Mehmi I et al Endogenous aryl hydrocarbon receptor promotes basal and inducible expression of tumor necrosis factor target genes in MCF-7 cancer cells. Biochem Pharmacol. 2014;91(3):390–399. doi: 10.1016/j.bcp.2014.06.015 24971714 10.1016/j.bcp.2014.06.015 PMC4157967

[50] Hollingshead BD, Beischlag TV, Dinatale BC, Ramadoss P, Perdew GH. Inflammatory signaling and aryl hydrocarbon receptor mediate synergistic induction of interleukin 6 in MCF-7 cells. Cancer Res. 2008;68(10):3609–3617. doi: 10.1158/0008-5472.CAN-07-6168 18483242 10.1158/0008-5472.CAN-07-6168 PMC2568985

[51] Murray IA, Krishnegowda G, DiNatale BC, Flaveny C, Chiaro C, Lin JM et al Development of a selective modulator of aryl hydrocarbon (Ah) receptor activity that exhibits anti-inflammatory properties. Chem Res Toxicol. 2010;23(5):955–966. doi: 10.1021/tx100045h 20423157 10.1021/tx100045h PMC2871980

[52] Pellikainen JM, Ropponen KM, Kataja VV, Kellokoski JK, Eskelinen MJ, Kosma VM. Expression of matrix metalloproteinase (MMP)-2 and MMP-9 in breast cancer with a special reference to activator protein-2, HER2, and prognosis. Clin Cancer Res. 2004;10(22):7621–7628. doi: 10.1158/1078-0432.CCR-04-1061 15569994 10.1158/1078-0432.CCR-04-1061

[53] Mountzios G, Aivazi D, Kostopoulos I, Kourea HP, Kouvatseas G, Timotheadou E et al Differential expression of the insulin-like growth factor receptor among early breast cancer subtypes. PLoS One. 2014;9(3):e91407 doi: 10.1371/journal.pone.0091407 24637962 10.1371/journal.pone.0091407 PMC3956672

[54] Litzenburger UM, Opitz CA, Sahm F, Rauschenbach KJ, Trump S, Winter M et al Constitutive IDO expression in human cancer is sustained by an autocrine signaling loop involving IL-6, STAT3 and the AHR. Oncotarget. 2014;5(4):1038–1051. doi: 10.18632/oncotarget.1637 24657910 10.18632/oncotarget.1637 PMC4011581

[55] Platten M, Wick W, Van den Eynde BJ. Tryptophan catabolism in cancer: beyond IDO and tryptophan depletion. Cancer Res. 2012;72(21):5435–5440. doi: 10.1158/0008-5472.CAN-12-0569 23090118 10.1158/0008-5472.CAN-12-0569

[56] Dere E, Forgacs AL, Zacharewski TR, Burgoon LD. Genome-wide computational analysis of dioxin response element location and distribution in the human, mouse, and rat genomes. Chem Res Toxicol. 2011;24(4):494–504. doi: 10.1021/tx100328r 21370876 10.1021/tx100328r PMC4038167

[57] Wormke M, Stoner M, Saville B, Safe S. Crosstalk between estrogen receptor alpha and the aryl hydrocarbon receptor in breast cancer cells involves unidirectional activation of proteasomes. FEBS Lett. 2000;478(1–2):109–112. 10922479 10.1016/s0014-5793(00)01830-5

[58] Jeffy BD, Hockings JK, Kemp MQ, Morgan SS, Hager JA, Beliakoff J et al An estrogen receptor-alpha/p300 complex activates the BRCA-1 promoter at an AP-1 site that binds Jun/Fos transcription factors: repressive effects of p53 on BRCA-1 transcription. Neoplasia. 2005;7(9):873–882. 16229810 10.1593/neo.05256 PMC1501940

[59] Hockings JK, Thorne PA, Kemp MQ, Morgan SS, Selmin O, Romagnolo DF. The ligand status of the aromatic hydrocarbon receptor modulates transcriptional activation of BRCA-1 promoter by estrogen. Cancer Res. 2006;66(4):2224–2232. doi: 10.1158/0008-5472.CAN-05-1619 16489025 10.1158/0008-5472.CAN-05-1619

[60] Tian Y, Ke S, Denison MS, Rabson AB, Gallo MA. Ah receptor and NF-kappaB interactions, a potential mechanism for dioxin toxicity. J Biol Chem. 1999;274(1):510–515. 9867872 10.1074/jbc.274.1.510

[61] Vogel CF, Sciullo E, Matsumura F. Involvement of RelB in aryl hydrocarbon receptor-mediated induction of chemokines. Biochem Biophys Res Commun. 2007;363(3):722–726. doi: 10.1016/j.bbrc.2007.09.032 17900530 10.1016/j.bbrc.2007.09.032 PMC4275028

[62] Safe S, Qin C, McDougal A. Development of selective aryl hydrocarbon receptor modulators for treatment of breast cancer. Expert Opin Investig Drugs. 1999;8(9):1385–1396. doi: 10.1517/13543784.8.9.1385 15992156 10.1517/13543784.8.9.1385

[63] Zhang S, Lei P, Liu X, Li X, Walker K, Kotha L et al The aryl hydrocarbon receptor as a target for estrogen receptor-negative breast cancer chemotherapy. Endocr Relat Cancer. 2009;16(3):835–844. doi: 10.1677/ERC-09-0054 19447902 10.1677/ERC-09-0054 PMC2766348

[64] Stark K, Burger A, Wu J, Shelton P, Polin L, Li J. Reactivation of estrogen receptor alpha by vorinostat sensitizes mesenchymal-like triple-negative breast cancer to aminoflavone, a ligand of the aryl hydrocarbon receptor. PLoS One. 2013;8(9):e74525 doi: 10.1371/journal.pone.0074525 24058584 10.1371/journal.pone.0074525 PMC3772827

[65] Jin UH, Lee SO, Pfent C, Safe S. The aryl hydrocarbon receptor ligand omeprazole inhibits breast cancer cell invasion and metastasis. BMC Cancer. 2014;14:498 doi: 10.1186/1471-2407-14-498 25011475 10.1186/1471-2407-14-498 PMC4226953

